# Shared genetic liability for alcohol consumption, alcohol problems, and suicide attempt: Evaluating the role of impulsivity

**DOI:** 10.1038/s41398-023-02389-3

**Published:** 2023-03-10

**Authors:** Mallory Stephenson, Séverine Lannoy, Alexis C. Edwards

**Affiliations:** Department of Psychiatry, Virginia Institute for Psychiatric and Behavioral Genetics, Virginia Commonwealth University, Richmond, VA USA

**Keywords:** Genetics, Psychology

## Abstract

Heavy drinking and diagnosis with alcohol use disorder (AUD) are consistently associated with risk for suicide attempt (SA). Though the shared genetic architecture among alcohol consumption and problems (ACP) and SA remains largely uncharacterized, impulsivity has been proposed as a heritable, intermediate phenotype for both alcohol problems and suicidal behavior. The present study investigated the extent to which shared liability for ACP and SA is genetically related to five dimensions of impulsivity. Analyses incorporated summary statistics from genome-wide association studies of alcohol consumption (*N* = 160,824), problems (*N* = 160,824), and dependence (*N* = 46,568), alcoholic drinks per week (*N* = 537,349), suicide attempt (*N* = 513,497), impulsivity (*N* = 22,861), and extraversion (*N* = 63,030). We used genomic structural equation modeling (Genomic SEM) to, first, estimate a common factor model with alcohol consumption, problems, and dependence, drinks per week, and SA included as indicators. Next, we evaluated the correlations between this common genetic factor and five factors representing genetic liability to negative urgency, positive urgency, lack of premeditation, sensation-seeking, and lack of perseverance. Common genetic liability to ACP and SA was significantly correlated with all five impulsive personality traits examined (*rs* = 0.24–0.53, *p*s < 0.002), and the largest correlation was with lack of premeditation, though supplementary analyses suggested that these findings were potentially more strongly influenced by ACP than SA. These analyses have potential implications for screening and prevention: Impulsivity can be comprehensively assessed in childhood, whereas heavy drinking and suicide attempt are quite rare prior to adolescence. Our findings provide preliminary evidence that features of impulsivity may serve as early indicators of genetic risk for alcohol problems and suicidality.

## Introduction

Alcohol use disorder (AUD) and suicide attempt (SA) represent substantial public health concerns. In the United States alone, the annual costs attributable to alcohol consumption and SA have been estimated as $234 billion [1] and $5.2 billion [2], respectively. Evidence suggests that alcohol consumption, alcohol problems, and SA are also related to one another: Heavy drinkers exhibit five times greater risk for suicide death when compared to social drinkers [3], and diagnosis with AUD is associated with increased risk for suicide attempt and death [4, 5], highlighting the importance of understanding the complex and potentially shared etiology between these phenotypes.

There are several possible explanations for the association between AUD and suicidal behavior. The relationship of alcohol consumption and problems (ACP) with SA may be causal, such that alcohol use increases risk for suicidal ideation and attempt, SA results in escalation of ACP, or both. Another possible, and non-mutually exclusive, explanation is that associations between ACP and suicidal behavior are non-causal, attributable to shared environmental factors and/or overlapping genetic influences. AUD and SA show substantial heritability [6–8] and are significantly genetically correlated [7, 9], supporting the presence of overlapping genetic influences between phenotypes. However, the shared genetic architecture of ACP and SA remains largely uncharacterized.

Only one study, to date, has evaluated potential explanations for the genetic overlap between substance use disorders (SUDs) and suicide-related outcomes [10]. In this work, Colbert and colleagues estimated the genetic correlations between a common SUD factor, which included opioid use disorder, cannabis use disorder, nicotine dependence, and problematic alcohol use as indicators, and suicide-related behaviors. The SUD factor was significantly correlated with SA, and the magnitude of this correlation decreased but remained statistically significant after including major depression and risk tolerance as covariates. Therefore, major depression and risk tolerance accounted for some, but not all, of the genetic correlation between SUDs and SA [10]. Nonetheless, additional work is needed to further understand the nature of shared genetic influences on alcohol-related outcomes and suicide attempt, as Colbert et al. focused on common genetic influences among SUDs and utilized a general measure of one’s willingness to take risks.

Identifying shared endophenotypes is one important step toward understanding the observed genetic correlation between alcohol-related outcomes and SA [11]. To be considered an endophenotype, a trait must be associated with risk for psychiatric disorder, be heritable, show state independence, co-segregate with the disorder within families, and be found in unaffected family members at a higher rate than in the general population [12]. The present study focused on impulsivity, which features prominently in theoretical models of suicidal behavior [13, 14] and has been proposed as a candidate endophenotype for both ACP [15] and suicidality [16]. Though the association between self-report measures of impulsivity and suicidal behavior is small in magnitude [17], impulsivity is moderately heritable [18] and is associated with elevated risk for alcohol problems and SA [19–22]. Impulsivity, AUD, and suicidality also tend to cluster together within individuals. For instance, one study conducted by Dumais et al. [23] compared individuals who died by suicide within the context of major depressive disorder (MDD) to living controls affected by MDD only. They found that individuals who died by suicide exhibited more impulsive behavior and were more likely to meet criteria for AUD when compared to the MDD-affected control group.

The extant evidence suggests that impulsivity meets at least some of the criteria for an endophenotype. However, when evaluating the relationships among impulsivity, ACP, and suicidal behavior, it is critical to consider what is meant by “impulsivity.” This term has been applied to a heterogeneous amalgamation of constructs related to rash action, behavioral disinhibition, novelty-seeking, and inattention. As a result, studies of “impulsivity” may actually be studying different constructs, which impedes our understanding of the associations among impulsivity, ACP, and suicidality.

Efforts to integrate varying conceptualizations of impulsivity [24–26] have provided evidence for five discrete personality traits underlying impulsive action (collectively referred to as the UPPS-P Model of Impulsive Personality): negative urgency, positive urgency, lack of premeditation, sensation-seeking, and lack of perseverance. Negative and positive urgency reflect the tendency to engage in rash action when experiencing negative or positive affect, respectively; lack of premeditation represents the tendency to act without careful thought and planning; sensation-seeking assesses the tendency to seek out excitement; and lack of perseverance measures the inclination to give up before completing a boring or difficult task. These impulsive personality traits map onto different facets of personality, such that positive and negative urgency are related to neuroticism, lack of premeditation and lack of perseverance are related to conscientiousness, and sensation-seeking is related to extraversion [24]. Further, they show varying patterns of association with risky behavior. For example, sensation-seeking is generally associated with the frequency of engaging in risky behaviors (e.g., binge drinking), whereas urgency is associated with problematic levels of involvement in risky behavior (e.g., AUD) [20, 25].

A recent genome-wide association study (GWAS) indicates that negative urgency, positive urgency, lack of premeditation, sensation-seeking, and lack of perseverance are partially distinct at the genetic level, as well. Sanchez-Roige et al. [27] conducted separate GWAS of sub-scores from the UPPS-P Impulsive Behavior Scale, which assesses the five impulsive personality traits proposed by the UPPS-P Model of Impulsive Personality. They found that the proportion of variance explained by single nucleotide polymorphisms (SNPs), or the SNP-based heritability, varied from 4.52% for lack of premeditation to 8.11% for sensation-seeking, and each impulsive personality trait showed different patterns of association with psychiatric disorders [27]. Moreover, a follow-up study performed by Gustavson et al. [28] provided evidence for five latent genetic factors underlying impulsivity, which correspond to the five impulsive personality traits proposed by the UPPS-P. Genetic correlations of negative urgency, positive urgency, lack of premeditation, sensation-seeking, and lack of perseverance with internalizing pathology ranged from −0.10 to 0.55, further underscoring the need to adopt a multidimensional approach when examining how impulsivity relates to ACP and SA.

The present study built upon genetic analyses of impulsive personality traits [27, 28] and methodological advances in analyzing the joint architecture of complex traits [29] to characterize the genetic relationships among ACP, SA, and impulsivity. A common genetic factor was first derived for alcohol consumption, problems, and dependence, as well as SA. A range of alcohol-related outcomes was included in this model, as alcohol consumption and problems are moderately genetically correlated [30] and are both associated with risk for suicidal behavior [3, 31]. The primary aim was to evaluate the degree to which this shared genetic liability for ACP and SA is related to the five impulsive personality traits proposed by the UPPS-P Model of Impulsive Personality [24]. Evidence suggests that impulsivity, alcohol use and problems, and suicidal behavior are related to one another [19–23, 32], and impulsivity has been proposed as an endophenotype for AUD [15] and suicidality [16]. However, “impulsivity” is not tractable as a shared endophenotype for ACP and SA because it is not a unitary construct. Therefore, by separately considering the genetic underpinnings of positive and negative urgency, lack of premeditation, sensation-seeking, and lack of perseverance, the present study provides insight into which impulsive personality traits (if any) are associated with shared genetic liability for ACP and SA and may thus serve as a candidate shared endophenotype.

## Materials and methods

### Samples

Analyses incorporated summary statistics from previously published GWAS of alcohol consumption [33, 34], problems [33], and dependence [35]; SA [7]; and impulsive personality traits [27, 36] (Table 1). Two measures of alcohol consumption—the Alcohol Use Disorder Identification Test—Consumption subscale (AUDIT-C) and drinks consumed per week—were used, as inclusion of multiple highly correlated phenotypes increases statistical power in Genomic SEM [29]. Further, the AUDIT-C uniquely assesses patterns of binge drinking, in addition to quantity and frequency of alcohol consumption. To remain consistent with prior work on the genetic structure of impulsivity [28], the UPPS-P Impulsive Behavior subscales, the Barratt Impulsiveness Scale (BIS), and extraversion were included as measures of impulsive personality traits.

**Table 1:** Descriptive statistics for genome-wide association studies of alcohol-related outcomes, suicide attempt, and impulsive personality traits.

Trait	Abbreviation	PMID	Sample Size	$h_{\rm{liab}}^2$ (SE)
Alcohol consumption	ALC	33985350	160,824	0.0729 (0.0047)
Alcohol problems	ALP	33985350	160,824	0.0372 (0.0037)
Alcohol dependence	DEP	30482948	46,568	0.1175 (0.0189)
Drinks per week	DPW	30643251	537,349	0.0492 (0.0022)
Suicide attempt	SA	34861974	513,497	0.0399 (0.0060)
Negative urgency	NEG	30718321	22,795	0.0799 (0.0218)
Positive urgency	POS	30718321	22,738	0.0682 (0.0219)
Lack of premeditation	MED	30718321	22,774	0.0402 (0.0195)
BIS total score	BIS	30718321	21,495	0.0617 (0.0209)
Sensation-seeking	SEN	30718321	22,745	0.0836 (0.0192)
Extraversion	EXT	26362575	63,030	0.0508 (0.0081)
Lack of perseverance	PER	30718321	22,861	0.0774 (0.0193)

*Notes*. Samples were limited to individuals of European ancestry, as genomic structural equation modeling requires summary statistics from ethnically homogeneous samples. Though genome-wide association studies of impulsive personality traits were conducted in the same sample, sample sizes differ based on the presence of complete data. *Abbreviations*. *PMID* PubMed identifier, $h_{\rm{liab}}^2$ = liability-scale heritability estimate, *SE* standard error.

#### Alcohol Use Disorder Identification Test (AUDIT)

Summary statistics for alcohol consumption and problems were from a GWAS meta-analysis conducted with three population-based cohorts (*N* = 160,824) [33]. Summary statistics were generated separately in each sample for each of the 10 AUDIT items, then item-level summary statistics were meta-analyzed across samples using METAL [37]. Next, multivariate GWAS analyses were performed by estimating SNP associations with two latent genetic factors. The consumption factor (AUDIT-C) included the first three items of the AUDIT, which evaluate participants’ frequency of alcohol consumption, quantity of alcohol consumption, and frequency of binge drinking (defined as 6+ drinks on one occasion). The problems factor (AUDIT-P) included the seven remaining items, which assess problematic alcohol use. The present analyses included summary statistics for both the AUDIT-C and AUDIT-P latent factors.

#### Alcoholic drinks per week

Summary statistics for drinks per week were from a GWAS meta-analysis performed by the GWAS and Sequencing Consortium of Alcohol and Nicotine use (GSCAN) [34]. Twenty-four studies with data on the average number of drinks consumed by participants each week were included in the analysis. Summary statistics were generated in each sample, then meta-analyzed using rareGWAMA [38]. The present analysis was limited to individuals of European ancestry, as Genomic SEM requires summary statistics from ethnically homogeneous samples [29]. In addition, data for 23andMe participants were not available, yielding a final sample size of 537,349 individuals.

#### Alcohol dependence

Summary statistics for alcohol dependence were from a GWAS meta-analysis of DSM-IV alcohol dependence conducted by the Psychiatric Genomics Consortium [35], which included data from 28 cohorts. Individuals who met DSM-IV criteria for alcohol dependence were defined as cases. When possible, individuals who had never tried alcohol or who met criteria for DSM-IV alcohol abuse were excluded as controls. Summary statistics were generated in each sample, then meta-analyzed using METAL [37]. Analyses were limited to 46,568 individuals of European ancestry (11,569 cases and 34,999 controls).

#### Suicide attempt (SA)

The International Suicide Genetics Consortium performed a GWAS meta-analysis of SA based on data from 18 cohorts [7]. Cases were individuals with a history of SA, defined as a lifetime act of deliberate self-harm with intent to die. Controls were individuals with no evidence of suicidal behavior. A separate GWAS was performed for each cohort, and summary statistics were meta-analyzed using METAL [37]. To address the presence of overlapping but distinct genetic influences on non-fatal suicide attempt and suicide death [39], the present study excluded cases who died by suicide. Therefore, analyses included 513,497 individuals of European ancestry (21,475 cases and 492,022 controls).

#### Impulsivity

Sanchez-Roige et al. [27] performed GWAS on multiple measures of impulsive personality traits collected by 23andMe, Inc., a consumer genetics company. Impulsivity was measured using the 20-item UPPS-P Impulsive Behavior Scale, which includes sub-scales for negative urgency, positive urgency, lack of premeditation, sensation-seeking, and lack of perseverance [24, 40]. Participants also completed the 30-item Barratt Impulsiveness Scale (BIS) [41]. Summary statistics for the BIS total score were included in the present analysis as an additional indicator of lack of premeditation, as the BIS total score and UPPS-P lack of premeditation are highly genetically correlated (*r*_g_ = 0.84) [27]. GWAS analyses were conducted using the 23andMe internal pipeline, with age, sex, five ancestral principal components, and genotype platform included as covariates [27]. Sample sizes ranged from 21,495 to 22,861 based on the presence of complete data.

#### Extraversion

Summary statistics for extraversion were from a GWAS meta-analysis conducted by the Genetics of Personality Consortium [36]. Within each of the 29 study samples (total *N* = 63,030), a latent extraversion score was calculated for each participant based on available extraversion item data [42]. Summary statistics were generated in each sample, then meta-analyzed using METAL [37].

### Statistical Methods

#### Conditional analysis

We conditioned SA summary statistics on the genetics of MDD because SA and MDD are highly genetically correlated (*r*_g_ = 0.78) [7], and MDD is prevalent among both AUD-affected individuals [43] and those who attempt or die by suicide [44]. Conditional summary statistics were generated using multi-trait-based conditional and joint analysis (mtCOJO) [45], implemented in GCTA [46]. This method uses genome-wide significant SNPs for the exposure trait (i.e., MDD) as instruments to estimate the effect of the exposure on the outcome, then conditions effect sizes and *p*-values based on the estimated effect of the exposure. We performed mtCOJO using the methodology detailed by Mullins et al. [7]. Summary statistics from the Wray et al. [47] GWAS of MDD were used for the exposure trait, and independent SNPs were selected as instruments if the *p*-value for their association with MDD was less than 5.0 × 10^–7^.

#### Preparing summary statistics

Next, we used the munge() function in the GenomicSEM R package [48] to pre-process summary statistics input files. During this step, SNPs were removed if they did not match the population reference set [49], had low imputation quality (INFO score < 0.90), or had a minor allele frequency (MAF) less than 0.01. For binary traits (i.e., alcohol dependence and SA), we computed effective sample sizes to correct for sample ascertainment. The effective sample size was calculated for each cohort using the formula *N*_eff_ = 4*υ*(1 − *υ*)*n*, where *υ* is the sample prevalence and *n* is the original sample size [50]. Effective sample sizes were then summed across cohorts. The total sample sizes were *N*_eff_ = 26,853 for alcohol dependence and *N*_eff_ = 74,887 for SA.

#### Descriptive statistics

After preparing the summary statistics files, we computed liability-scale heritability estimates and pairwise genetic correlations between traits using LD Score regression [51].

#### Primary analyses

We used Genomic SEM [29] to investigate the genetic relationships between common genetic liability for alcohol consumption, alcohol problems, and SA and multiple dimensions of impulsivity. First, we tested a common factor model, with AUDIT-C, drinks per week, AUDIT-P, alcohol dependence, and SA included as indicators of a single latent factor (denoted *C*_*g*_). A Comparative Fit Index (CFI) greater than 0.90 and Standardized Root Mean Squared Residual (SRMR) less than 0.10 were considered as evidence for acceptable model fit [52, 53].

Next, we performed a confirmatory factor model to reproduce a prior analysis of the genetic architecture of impulsivity [28], which provided evidence for five latent genetic factors underlying impulsivity based on the same set of summary statistics utilized in the present work. Factor loadings were equated for factors with only two indicators and fixed to 1.0 for factors with only one indicator. Finally, we specified a factor model to estimate the genetic correlations between a common genetic factor for alcohol consumption, alcohol problems, and SA (*C*_*g*_) and these five dimensions of impulsivity (negative urgency, positive urgency, lack of premeditation, sensation-seeking, and lack of perseverance). *P* < 0.05 was used as the threshold for statistical significance.

#### Secondary analysis: Genetic multivariable regression

To evaluate whether genetic correlations with impulsive personality traits were driven by variance specific to any one phenotype (i.e., AUDIT-C, drinks per week, AUDIT-P, alcohol dependence, or SA), we also conducted a series of genetic multivariable regression analyses [29]. Univariable summary statistics for each impulsivity-related construct were simultaneously regressed onto the genetic components of alcohol consumption, AUDIT-P, alcohol dependence, and SA. Because AUDIT-C and drinks per week were highly genetically correlated (*r*_*g*_ = 0.95), they were included as indicators of a latent genetic factor for alcohol consumption to avoid issues with multicollinearity. We specified separate models for UPPS-P negative urgency, UPPS-P positive urgency, UPPS-P lack of premeditation, UPPS-P sensation-seeking, UPPS-P lack of perseverance, the BIS total score, and extraversion.

## Results

### Heritability estimates and genetic correlations

**Fig. 1:**
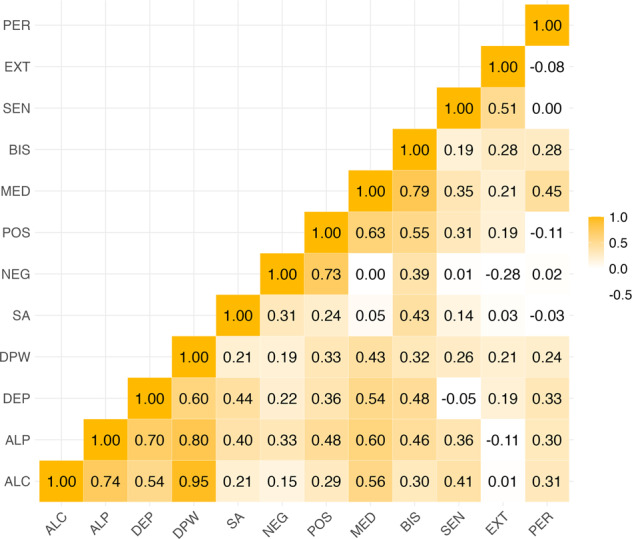
Pairwise genetic correlations between alcohol consumption and problems, suicide attempt, and impulsive personality traits. Standard errors can be found in Table S1.

Liability-scale heritability estimates are presented in Table 1, and Fig. 1 shows the pairwise genetic correlations estimated by LDSC (see Table S1 for standard errors). AUDIT-C, AUDIT-P, alcohol dependence, and drinks per week exhibited moderate to strong correlations with one another (*r*_*g*_ = 0.54–0.95, SE = 0.05–0.08). Among the alcohol-related outcomes, suicide attempt exhibited the highest genetic correlation with alcohol dependence (*r*_*g*_ = 0.44, SE = 0.11). Genetic correlations among impulsive personality traits ranged from *r*_*g*_ = −0.28 (SE = 0.15) for negative urgency and extraversion to *r*_*g*_ = 0.79 (SE = 0.35) for lack of premeditation and the BIS total score, underscoring that the dimensions of impulsive personality proposed by the UPPS-P have distinct genetic underpinnings.

### Genomic structural equation modeling

We evaluated the genetic overlap between shared genetic liability for ACP and SA and impulsive personality traits using Genomic SEM. As a preliminary step, we estimated a common factor model, with AUDIT-C, drinks per week, AUDIT-P, alcohol dependence, and SA included as indicators of a single latent factor (*C*_*g*_). A common factor model provided good fit to the data (χ^2^(5) = 33.82, *p* < 0.0001, AIC = 53.82, CFI = 0.980, SRMR = 0.095). SA exhibited the lowest loading on the common genetic factor (*λ* = 0.27, SE = 0.04, *p* < 0.0001). However, the factor loading for SA was statistically significant, suggesting that a significant proportion of the genetic variance in SA is accounted for by *C*_*g*_. The factor loadings were estimated as 0.93 (SE = 0.04, *p* < 0.0001) for AUDIT-C, 0.84 (SE = 0.05, *p* < 0.0001) for AUDIT-P, 0.65 (SE = 0.05, *p* < 0.0001) for alcohol dependence, and 0.97 (SE = 0.04, *p* < 0.0001) for drinks per week (Fig. S1).

**Fig. 2:**
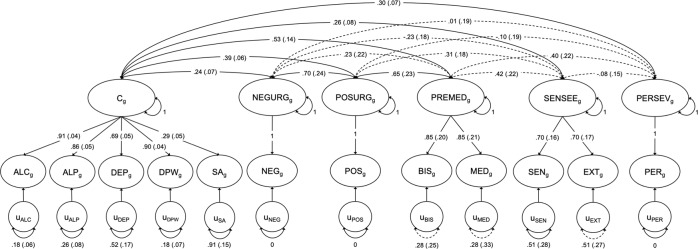
Genetic associations between common liability for alcohol consumption, alcohol problems, and suicide attempt and impulsive personality traits. Parameter estimates were fully standardized; standard errors are shown in parentheses. Solid lines denote statistically significant paths (*p* < 0.05) and fixed paths. Dashed lines represent non-significant paths. For factors with only one indicator, factor loadings were fixed to 1, and the residual variance of the indicator was fixed to 0. Loadings were equated for factors with only two indicators.

Next, we performed a confirmatory factor model to reproduce the five-factor model of impulsivity from Gustavson et al. [28]. As shown in Fig. S2, parameter estimates were reproduced within a margin of error of ±0.06. Model fit indices were also comparable and suggested that a five-factor model provided good fit to the data (χ^2^(9) = 7.55, *p* = 0.5800, AIC = 45.55, CFI = 1.000, SRMR = 0.072). We then specified the primary model (χ^2^(44) = 144.58, *p* < 0.0001, AIC = 212.58, CFI = 0.965, SRMR = 0.105), which estimated genetic correlations between *C*_*g*_, negative urgency, positive urgency, lack of premeditation, sensation-seeking, and lack of perseverance. Though the SRMR was greater than 0.10, the high CFI value provided evidence to support adequate model fit. In this model, shared genetic liability to ACP and SA was genetically correlated with all five dimensions of impulsivity. *C*_*g*_ was most highly correlated with lack of premeditation, followed by positive urgency, lack of perseverance, sensation-seeking, and negative urgency (Fig. 2).

### Supplementary analyses

We conducted two supplementary analyses to further explore findings from our primary model. First, because SA exhibited a low factor loading on *C*_*g*_, we conducted a follow-up analysis to test whether the observed genetic correlations between *C*_*g*_ and impulsive personality traits are solely driven by genetic variance specific to ACP. In this model, C_g_ was supplanted by an alcohol-specific latent factor (ACP_g_), with AUDIT-C, drinks per week, AUDIT-P, and alcohol dependence included as indicators, and a suicide-specific latent factor (SUIC_g_), with SA included as a single indicator. This alternative model also provided good fit to the data (*χ*^2^(39) = 142.86, *p* < 0.0001, AIC = 220.86, CFI = 0.964, SRMR = 0.099). Parameter estimates are shown in Fig. S3. The genetic correlations of ACP_g_ with negative urgency, positive urgency, lack of premeditation, sensation-seeking, and lack of perseverance were very similar, but did differ slightly, from the correlations between *C*_*g*_ and impulsive personality traits in the primary model. The suicide-specific latent factor was most highly correlated with lack of premeditation (*r*_*g*_ = 0.31, SE = 0.18) and negative urgency (*r*_*g*_ = 0.31, SE = 0.17), though these associations did not reach statistical significance.

Second, because SA was more highly genetically correlated with indices of alcohol problems (*r*_*g*_ = 0.40–0.44) than consumption (*r*_*g*_ = 0.21–0.21), we specified a follow-up model with AUDIT-C and drinks per week removed from the common genetic factor. This model provided good fit to the data (*χ*^2^(25) = 50.32, *p* = 0.0002, AIC = 110.32, CFI = 0.940, SRMR = 0.091). Compared to *C*_*g*_ in the primary model, a common genetic factor for alcohol problems and SA was more highly correlated with negative urgency (*r*_*g*_ = 0.36 versus 0.24), positive urgency (*r*_*g*_ = 0.50 versus 0.39), lack of premeditation (*r*_*g*_ = 0.68 versus 0.53), and lack of perseverance (*r*_*g*_ = 0.32 versus 0.30). Conversely, the correlation with sensation-seeking was smaller in magnitude (*r*_*g*_ = 0.14 versus 0.26) and statistically non-significant (Fig. S4).

### Genetic multivariable regression models

**Fig. 3:**
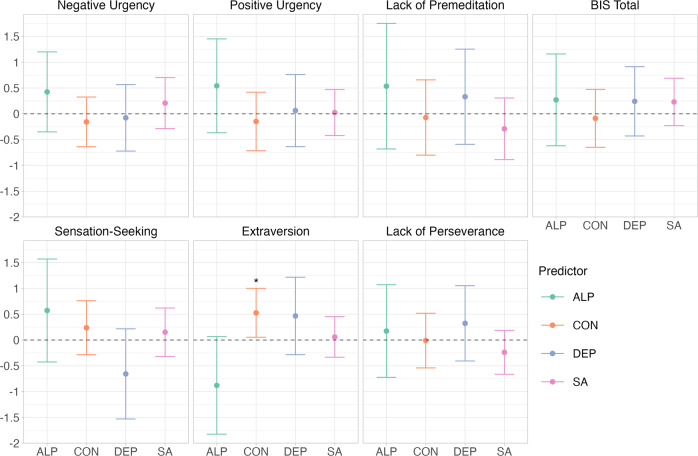
Standardized conditional associations of alcohol consumption, problems, and dependence and suicide attempt with impulsive personality traits. Each impulsive personality trait was considered in a separate model. $\hat \beta$ estimates are shown on the y-axis. Error bars represent 95% confidence intervals. Statistically significant parameter estimates are annotated with an asterisk.

Finally, to provide further insight into the genetic correlations of ACP and SA with impulsive personality traits, we specified seven genetic multivariable regression models. We included the genetic components of alcohol consumption (represented by a latent factor with AUDIT-C and drinks per week as indicators), AUDIT-P, alcohol dependence, and SA as predictors, and alternately treated the genetic components of UPPS-P negative urgency, UPPS-P positive urgency, UPPS-P lack of premeditation, BIS total score, UPPS-P sensation-seeking, extraversion, and UPPS-P lack of perseverance as the outcome. As shown in Fig. 3 (see Figs. S5–S11 for path diagrams), the conditional standardized associations of ACP and SA with impulsive personality traits were largely non-significant, with 95% confidence intervals overlapping zero.

## Discussion

In the current study, we investigated associations between shared genetic liability for ACP and SA and multiple correlated dimensions of impulsivity. A common genetic factor for alcohol consumption, alcohol problems, and suicidality was positively correlated with all five impulsive personality traits examined. However, the magnitude of these associations varied, such that shared genetic liability for ACP and SA was most strongly correlated with lack of premeditation. Our findings are consistent with prior work supporting shared genetic influences across ACP and suicidality [7, 10, 54]. In addition, we present evidence for genetic overlap between ACP, SA, and impulsive personality traits, providing further insight into previously established phenotypic associations [19–22, 55].

One question motivating the present work was whether impulsive personality traits may be considered as candidate shared endophenotypes for ACP and SA. Our findings suggest that shared genetic liability for ACP and SA overlaps with all five traits proposed by the UPPS-P Model of Personality: [24, 26] negative urgency, positive urgency, lack of premeditation, sensation-seeking, and lack of perseverance. A common genetic factor for ACP and SA showed a sizeable genetic correlation with lack of premeditation, suggesting that the tendency to engage in little forethought and planning may be of particular interest when considering the shared etiology of ACP and SA. These findings have potential implications for screening, prevention, and intervention. For instance, we provide very preliminary evidence that early assessment of impulsive personality traits may help identify individuals at risk for the later development of ACP and SA. Further, the cognitive mechanisms underlying lack of premeditation—risky decision-making and delay discounting [56, 57]—are modifiable and improve with neuropsychological intervention [58, 59], raising the possibility that intervening on risky decision-making and delay discounting could reduce risk for ACP and SA.

However, because the present analyses focused only on the genetic relationships among ACP, SA, and impulsive personality traits, additional work is needed before recommendations for prevention and intervention are warranted. First, studies should confirm that shared genetic liability for ACP and SA is associated with phenotypic levels of impulsivity across development. Second, further research is needed to distinguish whether the associations between ACP, SA, and impulsive personality traits are consistent with an endophenotype mediational model or an endophenotype liability-index model. Endophenotype mediational models propose that genetic influences on a trait or disorder are partially or fully mediated through the endophenotype, whereas endophenotype liability-index models suggest that a common set of genes are associated with the endophenotype and the disorder [60]. Importantly, intervention efforts to reduce impulsivity will only impact later risk for ACP and SA if associations are consistent with an endophenotype mediational model.

We also specified an alternative model in which common genetic liability for alcohol consumption, alcohol problems, and SA (*C*_*g*_) was replaced by an alcohol-specific latent factor (ACP_g_) and a suicide-specific latent factor (SUIC_g_). As observed for *C*_*g*_ in the primary analysis, ACP_g_ was significantly genetically correlated with all five impulsive personality traits. Conversely, none of the genetic correlations between SUIC_g_ and facets of impulsivity were statistically significant. In addition, ACP_g_ and SUIC_g_ differed somewhat in their genetic associations with features of impulsivity. For example, genetic liability for ACP was more strongly correlated with lack of perseverance and sensation-seeking, whereas genetic liability for SA showed a larger genetic correlation with negative urgency. This supplementary analysis suggests that results from our primary model are potentially more strongly influenced by ACP than SA.

We further explored our findings by conducting a follow-up analysis with AUDIT-C and drinks per week removed from the common genetic factor. Genetic correlations of shared liability for alcohol problems and SA with impulsive personality traits were even larger than observed for *C*_*g*_ in the primary model, except that the correlation with sensation-seeking was smaller in magnitude and statistically non-significant. This shift in parameter estimates likely reflects the overlapping, but partially distinct, genetic etiology of alcohol use versus AUD [30]. Nonetheless, taken together, these complementary approaches again suggest that genetic variance shared by alcohol outcomes and SA is positively correlated with facets of impulsivity, to varying degrees.

Finally, though our primary aim was to characterize associations of overlapping genetic influences on ACP and SA with negative urgency, positive urgency, lack of premeditation, sensation-seeking, and lack of perseverance, we also performed genetic multivariable regression analyses to contextualize the primary results and further describe the genetic relationships among ACP, SA, and impulsivity. Our primary factor model and the genetic multivariable regression models address complementary research questions: The main analyses assessed the degree to which *shared* genetic liability for ACP and SA is related to multiple dimensions of impulsivity, whereas genetic multivariable regression evaluated associations between each predictor (alcohol consumption, problems, and dependence and SA) and an impulsivity-related construct *unique* of the other predictors in the model.

In these analyses, the conditional standardized associations of ACP and SA with impulsive personality traits were largely non-significant, underscoring that the genetic relationships with impulsive personality traits are driven by genetic factors shared by ACP and SA. Nonetheless, some tentative insights may be drawn based on the magnitude of the associations. For example, the conditional standardized associations of alcohol consumption and dependence with negative urgency were small and negative ($\hat \beta _g$ = −0.16–0.08, SE = 0.25–0.33), whereas there were larger, but not statistically significant, associations of AUDIT-P and SA with negative urgency ($\hat \beta _g$ = 0.21–0.43, SE = 0.25–0.40). Genetic correlations with negative urgency may thus be primarily driven by genetic variance specific to AUDIT-P and SA. These findings suggest that impulsive personality traits may play a role in both the shared genetic etiology of ACP and SA and in their unique genetic etiology. However, conclusions should be considered preliminary, particularly in view of the large standard errors of the parameter estimates.

These analyses contribute to the extant literature in novel ways. We employed a recent methodological advance, Genomic SEM [29], to directly assess the extent to which genetic liability for impulsivity is correlated with genetic variance common to ACP and SA. In addition, we leveraged the availability of genetic findings on facets of impulsivity [27, 28] to determine which impulsive personality traits are associated with shared genetic risk for ACP and SA. This approach represents an alternative to studies that have operationalized impulsivity in a narrow manner (e.g., risk-taking behavior) and those that have collapsed this heterogeneous construct into a single measure.

However, our findings should also be considered in light of several limitations. First, the largest available GWAS of impulsive personality traits had a relatively small sample size (*N* = 22,861), which limited our statistical power and contributed to large 95% confidence intervals. Second, because Genomic SEM requires ancestrally homogeneous samples [29], and the vast majority of genome-wide studies are conducted in individuals of European descent [61], our analyses were limited to European ancestry individuals. Continued efforts to improve the representation of diverse populations in genetic research may facilitate replication of these analyses in other ancestral groups and increase the generalizability of findings. Third, in the GWAS of SA conducted by Mullins et al. [7], the control group was not limited to individuals with a history of suicidal ideation. As a result, summary statistics may, in part, capture genetic influences on suicidal ideation. However, SA summary statistics were conditioned on the genetics of MDD, which may partially address this concern.

Fourth, the present study focused on self-report measures of impulsive personality traits, though laboratory tasks are also widely used to assess impulsivity-related constructs. The correlations between self-report and task-based measures of impulsivity are small, and self-report scales and laboratory tasks are uniquely associated with risk behaviors [62]. Therefore, examining genetic relationships between shared liability for ACP and SA and performance on impulsivity-related laboratory tasks may be an important area for future work, particularly because laboratory tasks index specific cognitive processes related to impulsive behavior (e.g., delay discounting, response inhibition), which may be more tractable as an endophenotype.

Fifth, the present analyses focused on genetic liability for alcohol consumption and problems. Use and dependence on tobacco, cannabis, and other illicit substances have also been associated with risk for suicidality [63–65]. Though we anticipate that a similar pattern of associations would be observed if liability to other substance use and problems, or a general dimension of liability to substance involvement or addiction [66–68], were included in place of ACP, replicating these analyses with other substance use outcomes may be of interest to future researchers. Finally, our findings do not exclude the possibility of a causal relationship between ACP and SA. Investigating the joint roles of shared genetic etiology and causal mechanisms in the co-occurrence of alcohol-related outcomes and suicidality is an important next step.

In summary, we found that shared genetic liability among alcohol consumption, alcohol problems, and SA is positively correlated with the genetic components of multiple dimensions of impulsivity. These analyses have potential implications for screening and prevention: Impulsivity can be comprehensively assessed in childhood [69–71], whereas heavy drinking and SA are rare prior to adolescence [72, 73]. Therefore, our findings provide preliminary evidence that impulsive personality traits may serve as an early indicator of genetic risk for alcohol problems and suicidality. However, it will be important for future studies to establish that features of impulsivity are situated within the causal pathway from genetic variation to alcohol problems and suicide attempt in order to inform the development of effective interventions.

## Supplementary information

Supplementary Information

## Data Availability

Analysis scripts are available upon request.
