# Supplementary material for: Shared genetic liability for alcohol consumption, alcohol problems, and suicide attempt: Evaluating the role of impulsivity
Source: Transl Psychiatry. 2023 Mar 10;13:87. doi: 10.1038/s41398-023-02389-3 (PMC10006209; doi:10.1038/s41398-023-02389-3)
Supplement: Supplementary file 1 — Supplementary Information [file 41398_2023_2389_MOESM1_ESM.docx]

**Supplementary Information:** Shared Genetic Liability for Alcohol Consumption, Alcohol Problems, and Suicide Attempt: Evaluating the Role of Impulsivity

Table S1. Pairwise genetic correlations among alcohol-related outcomes, suicide attempt, and impulsive personality traits.

|  | **ALC** | **ALP** | **DEP** | **DPW** | **SA** | **NEG** | **POS** | **MED** | **BIS** | **SEN** | **EXT** | **PER** |
| --- | --- | --- | --- | --- | --- | --- | --- | --- | --- | --- | --- | --- |
| **ALC** | 1.000 |  |  |  |  |  |  |  |  |  |  |  |
| **ALP** | **0.738**  **(0.064)** | 1.000 |  |  |  |  |  |  |  |  |  |  |
| **DEP** | **0.544**  **(0.075)** | **0.701**  **(0.101)** | 1.000 |  |  |  |  |  |  |  |  |  |
| **DPW** | **0.947**  **(0.049)** | **0.796**  **(0.056)** | **0.604 (0.057)** | 1.000 |  |  |  |  |  |  |  |  |
| **SA** | **0.206**  **(0.062)** | **0.398**  **(0.086)** | **0.439 (0.109)** | **0.205 (0.051)** | 1.000 |  |  |  |  |  |  |  |
| **NEG** | 0.150  (0.087) | **0.329**  **(0.104)** | 0.218 (0.139) | **0.189 (0.071)** | 0.310 (0.175) | 1.000 |  |  |  |  |  |  |
| **POS** | **0.294 (0.092)** | **0.478**  **(0.117)** | **0.365 (0.154)** | **0.328 (0.072)** | 0.236 (0.149) | **0.732 (0.241)** | 1.000 |  |  |  |  |  |
| **MED** | **0.557 (0.128)** | **0.595**  **(0.160)** | **0.536 (0.203)** | **0.435 (0.092)** | 0.053 (0.196) | -0.002 (0.238) | **0.632 (0.273)** | 1.000 |  |  |  |  |
| **BIS** | **0.295 (0.092)** | **0.462**  **(0.115)** | **0.480 (0.156)** | **0.323 (0.076)** | **0.426 (0.164)** | 0.390 (0.237) | **0.545 (0.252)** | **0.794 (0.348)** | 1.000 |  |  |  |
| **SEN** | **0.406 (0.090)** | **0.361**  **(0.106)** | -0.047 (0.145) | **0.259 (0.077)** | 0.141 (0.148) | 0.011 (0.196) | 0.312 (0.193) | 0.350 (0.248) | 0.191 (0.218) | 1.000 |  |  |
| **EXT** | 0.010 (0.071) | -0.111  (0.086) | 0.192 (0.127) | **0.206 (0.063)** | 0.026 (0.115) | -0.279 (0.150) | 0.185 (0.146) | 0.213 (0.207) | 0.276 (0.160) | **0.513 (0.152)** | 1.000 |  |
| **PER** | **0.307 (0.096)** | **0.297**  **(0.121)** | **0.334 (0.150)** | **0.237 (0.073)** | -0.029 (0.146) | 0.018 (0.196) | -0.106 (0.197) | 0.446 (0.272) | 0.282 (0.218) | -0.003 (0.172) | -0.083 (0.130) | 1.000 |

*Notes.* Statistically significant correlations are shown in bold font. *Abbreviations.* ALC = alcohol consumption; ALP = alcohol problems; DEP = alcohol dependence; DPW = drinks per week; SA = suicide attempt; NEG = negative urgency; POS = positive urgency; MED = lack of premeditation; BIS = Barratt Impulsiveness Scale total score; SEN = sensation-seeking; EXT = extraversion; PER = lack of perseverance.


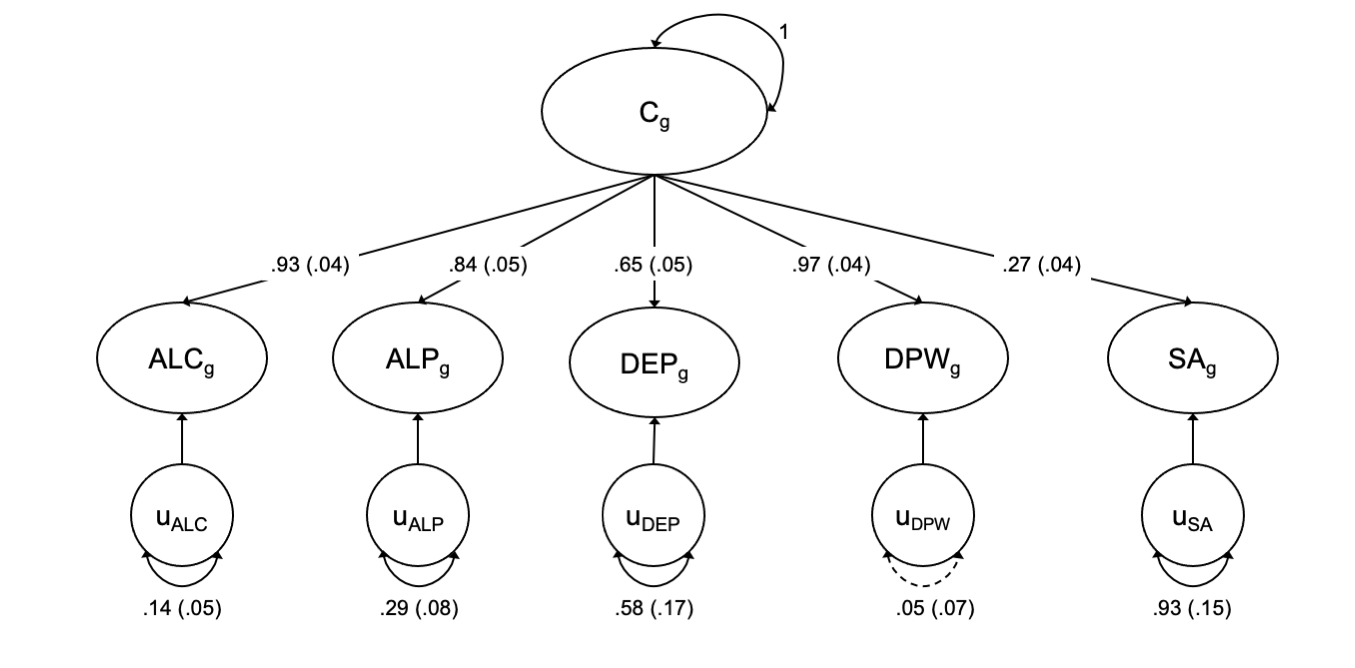


*Figure S1.* Common factor model for alcohol consumption, alcohol problems, and suicide attempt. Parameter estimates were standardized. Standard errors are shown in parentheses. Solid lines denote statistically significant paths (*p* < .05), and dashed lines represent non-significant paths.


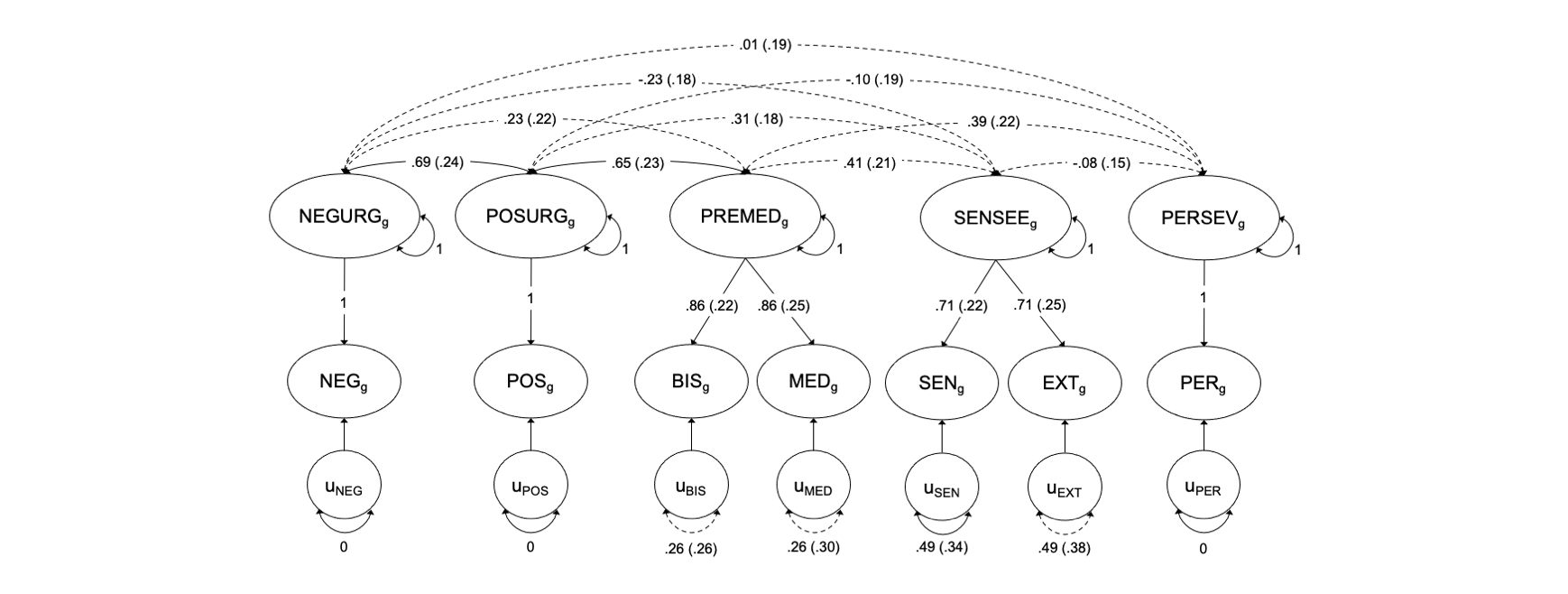
*Figure S2.* Confirmatory factor model for the genetic structure of impulsive personality traits. Parameter estimates were comparable to those in Gustavson et al. (PMID: 32716714) within a margin of $\pm$0.06. Parameter estimates were fully standardized; standard errors are shown in parentheses. Solid lines denote statistically significant paths (*p* < .05) and fixed paths. Dashed lines represent non-significant paths. For factors with only one indicator, factor loadings were fixed to 1, and the residual variance of the indicator was fixed to 0. Loadings were equated for factors with only two indicators.

*
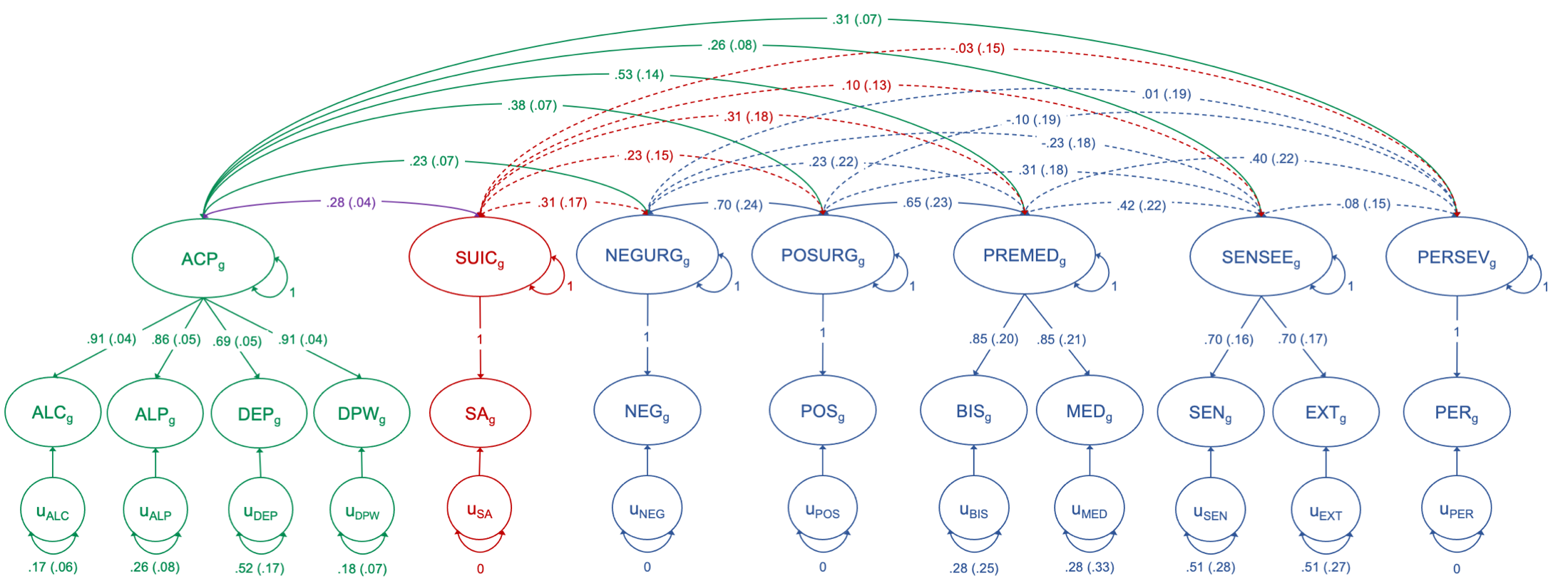
Figure S3.* Alternative model describing the genetic relationships among alcohol consumption and problems, suicide attempt, and impulsive personality traits. Parameter estimates were fully standardized; standard errors are shown in parentheses. Solid lines denote statistically significant paths (*p* < .05) and fixed paths. Dashed lines represent non-significant paths. The genetic correlations between alcohol consumption and problems and impulsive personality traits are shown in green, genetic correlations between suicide attempt and impulsive personality traits are shown in red, and genetic correlations among impulsive personality traits are shown in blue. The correlation between latent genetic liability for alcohol-related outcomes and suicide attempt is shown in purple. For factors with only one indicator, factor loadings were fixed to 1, and the residual variance of the indicator was fixed to 0. Loadings were equated for factors with only two indicators.

*
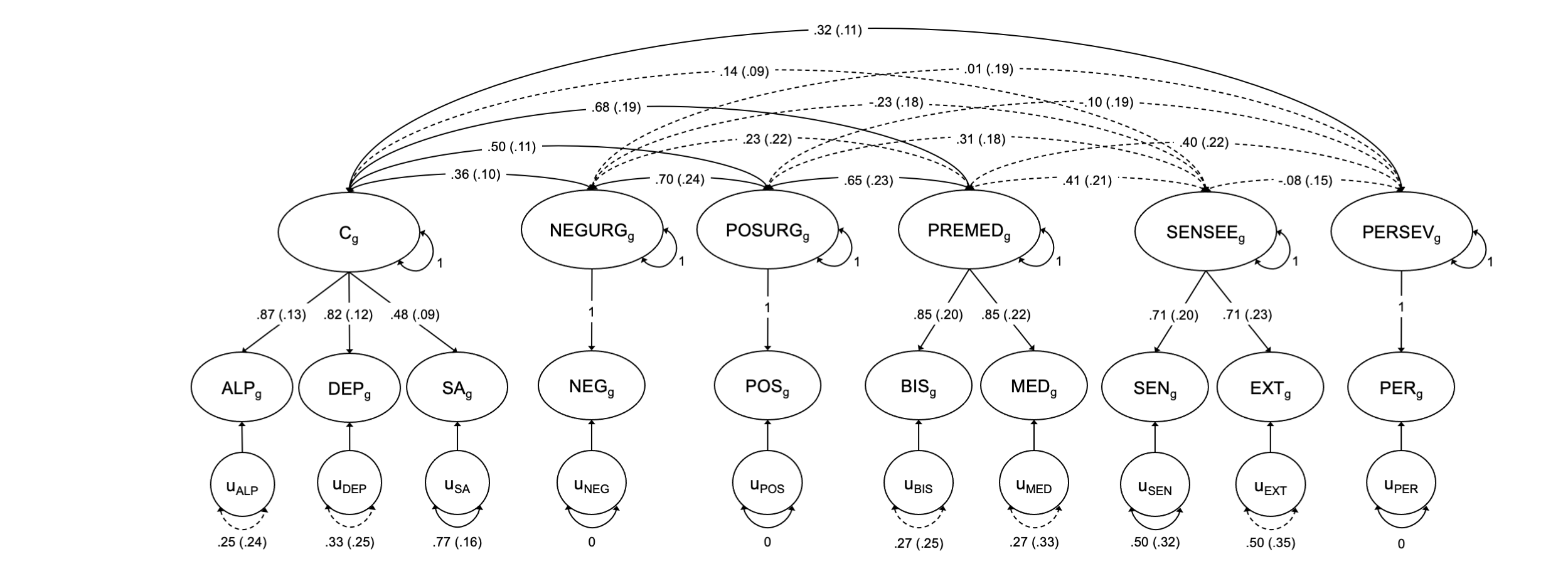
*

*Figure S4.* Alternative model showing the genetic associations of common liability for alcohol problems and suicide attempt with impulsive personality traits. Parameter estimates were fully standardized; standard errors are shown in parentheses. Solid lines denote statistically significant paths (*p* < .05) and fixed paths. Dashed lines represent non-significant paths. For factors with only one indicator, factor loadings were fixed to 1, and the residual variance of the indicator was fixed to 0. Loadings were equated for factors with only two indicators.


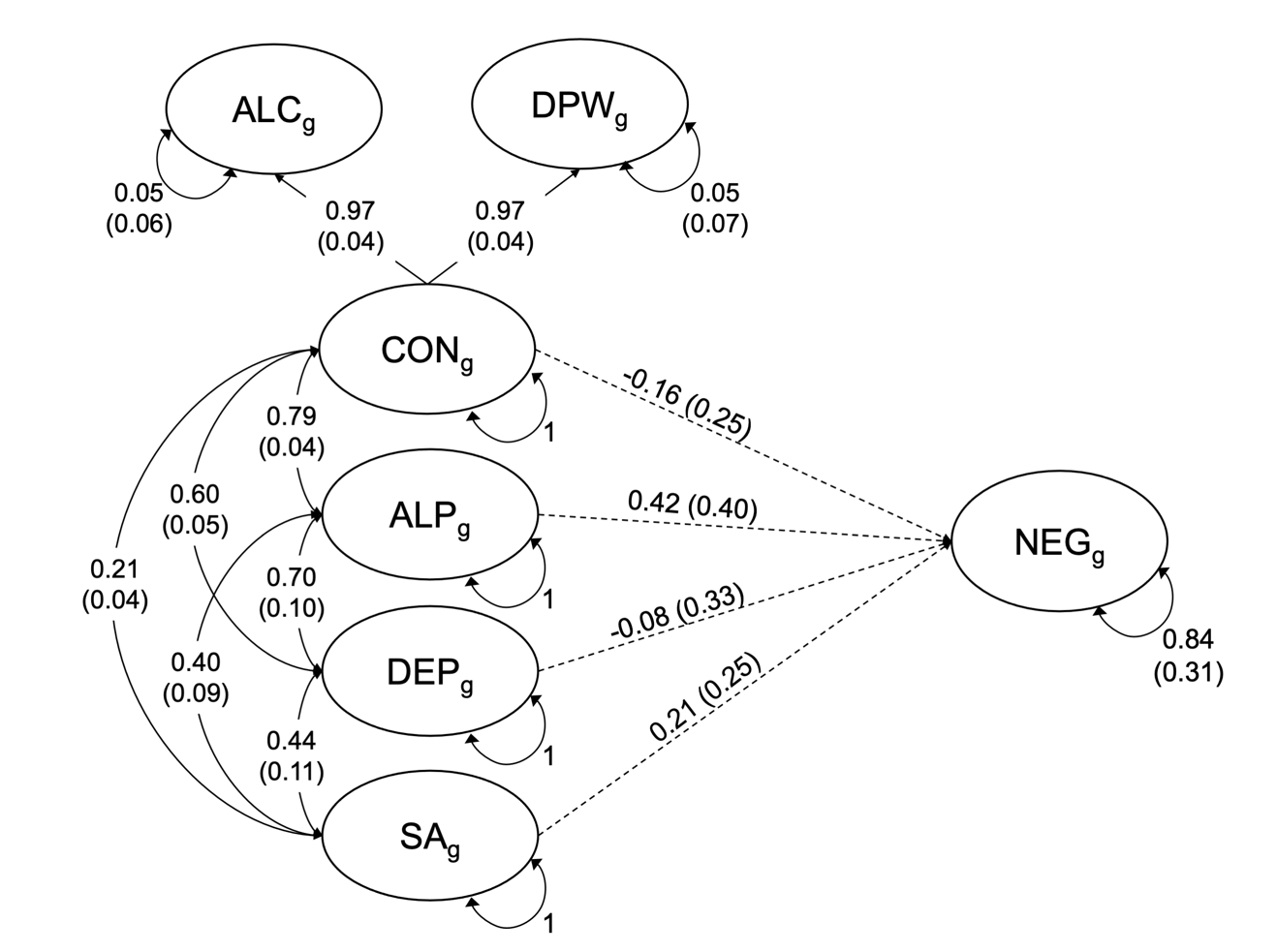


*Figure S5.* Genetic multivariable regression model with negative urgency. Parameter estimates were standardized. Standard errors are shown in parentheses. Solid lines denote statistically significant paths (*p* < .05), and dashed lines represent non-significant paths.


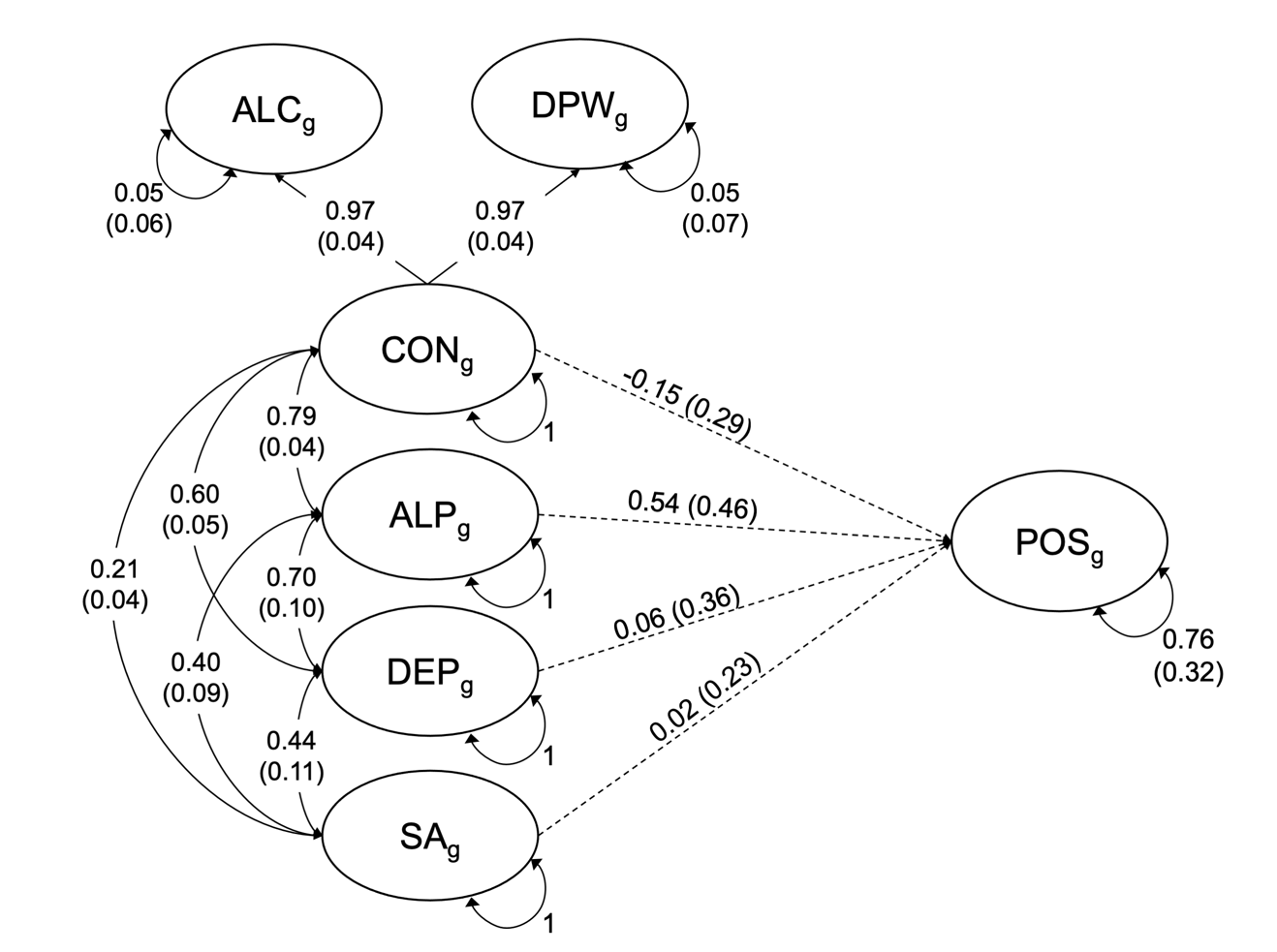


*Figure S6.* Genetic multivariable regression model with positive urgency. Parameter estimates were standardized. Standard errors are shown in parentheses. Solid lines denote statistically significant paths (*p* < .05), and dashed lines represent non-significant paths.


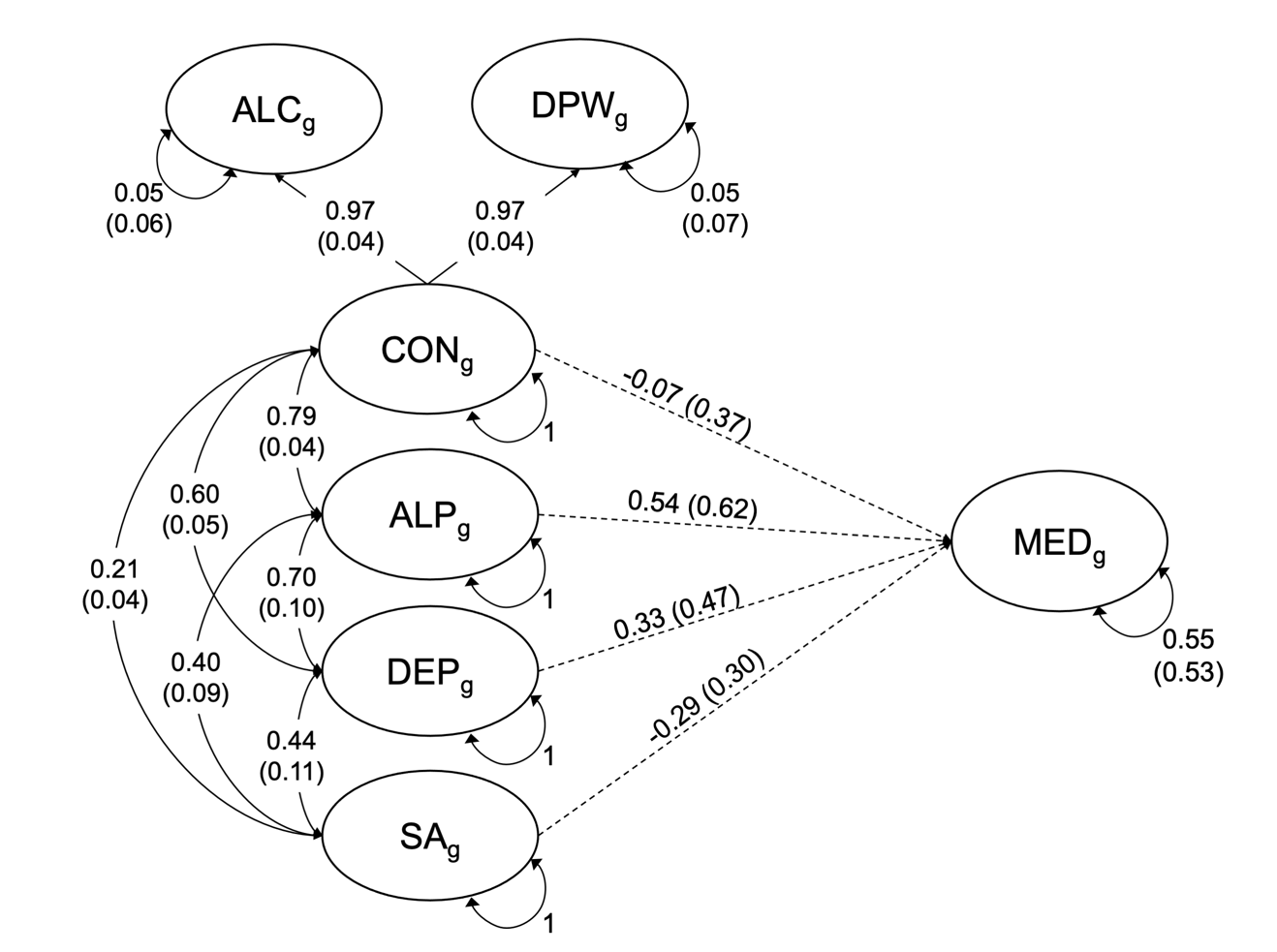


*Figure S7.* Genetic multivariable regression model with lack of premeditation. Parameter estimates were standardized. Standard errors are shown in parentheses. Solid lines denote statistically significant paths (*p* < .05), and dashed lines represent non-significant paths.


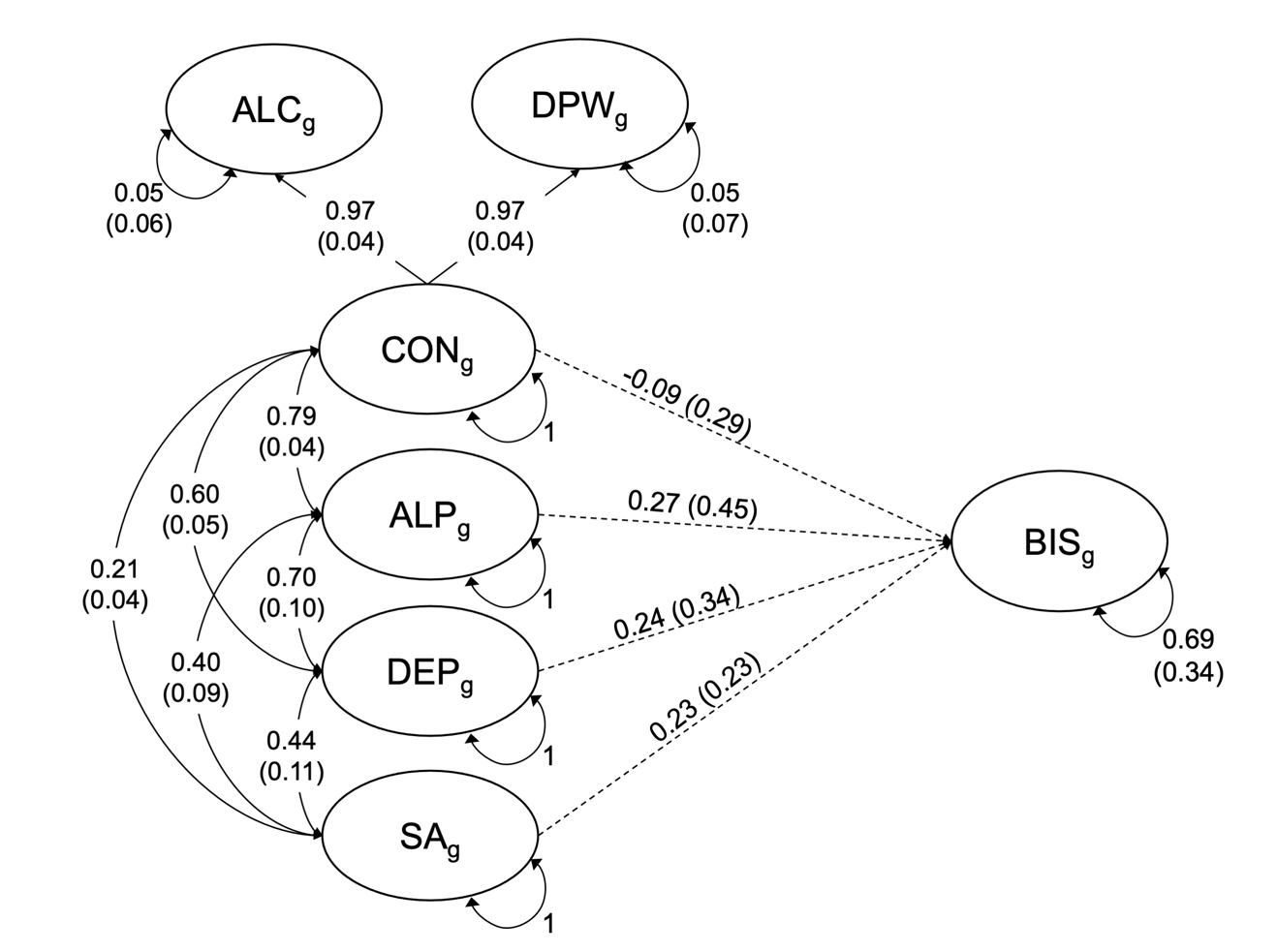


*Figure S8.* Genetic multivariable regression model with the Barratt Impulsiveness Scale total score. Parameter estimates were standardized. Standard errors are shown in parentheses. Solid lines denote statistically significant paths (*p* < .05), and dashed lines represent non-significant paths.


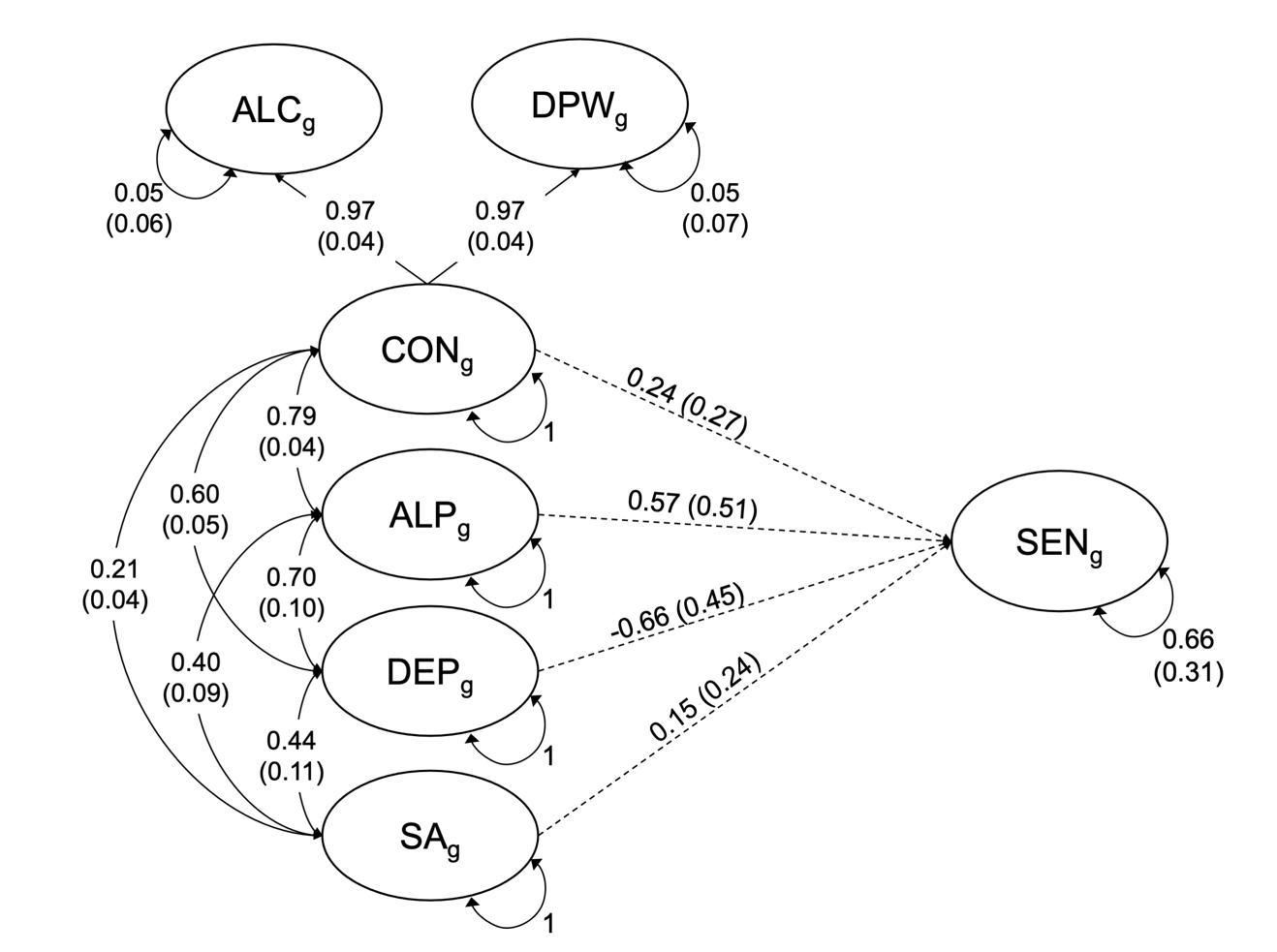


*Figure S9.* Genetic multivariable regression model with sensation-seeking. Parameter estimates were standardized. Standard errors are shown in parentheses. Solid lines denote statistically significant paths (*p* < .05), and dashed lines represent non-significant paths.


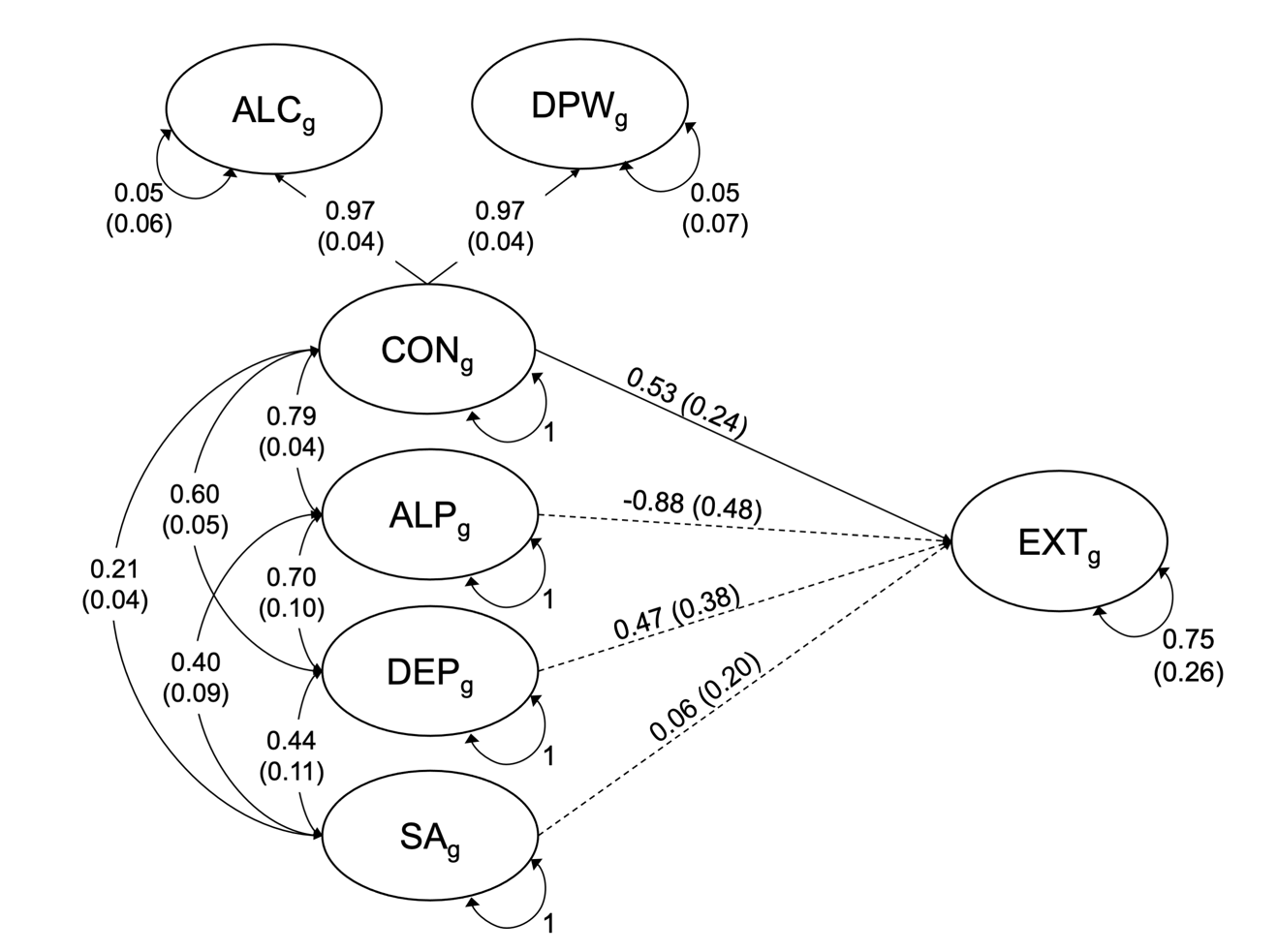


*Figure S10.* Genetic multivariable regression model with extraversion. Parameter estimates were standardized. Standard errors are shown in parentheses. Solid lines denote statistically significant paths (*p* < .05), and dashed lines represent non-significant paths.


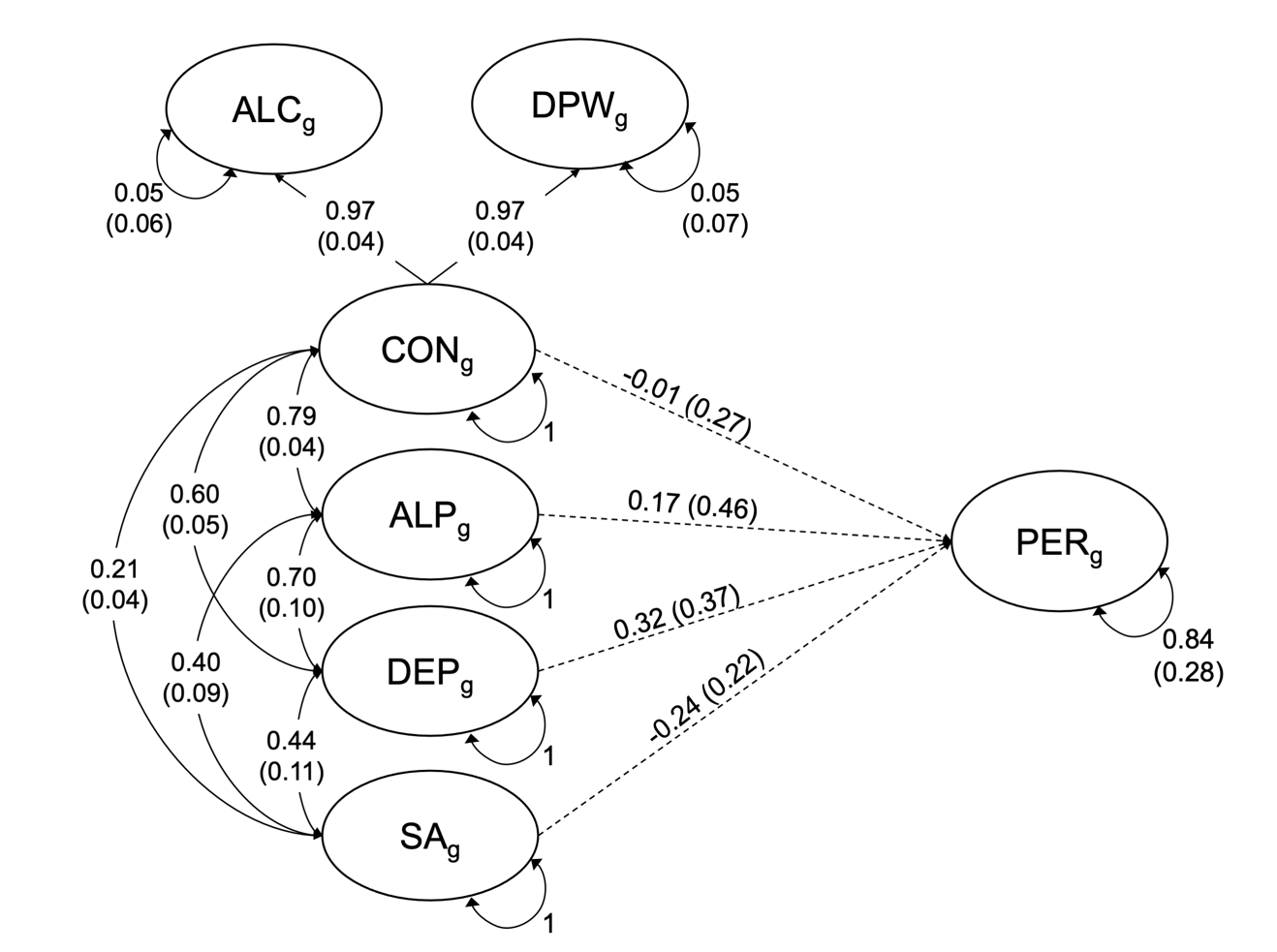


*Figure S11.* Genetic multivariable regression model with lack of perseverance. Parameter estimates were standardized. Standard errors are shown in parentheses. Solid lines denote statistically significant paths (*p* < .05), and dashed lines represent non-significant paths.
